# Exploring the Chemopreventive Potential of *Artemisia annua* Methanolic Extract in Colorectal Cancer Induced by Azoxymethane in Mice

**DOI:** 10.3390/ph18010034

**Published:** 2024-12-31

**Authors:** Faris Alrumaihi

**Affiliations:** Department of Medical Laboratories, College of Applied Medical Sciences, Qassim University, Buraydah 51452, Saudi Arabia; f_alrumaihi@qu.edu.sa; Tel.: +966-555181862; Fax: +966-6163801628

**Keywords:** natural products, artemisia extracts, colorectal cancer (CRC), alternative medicine

## Abstract

**Background/Objectives:** Colorectal cancer (CRC) remains a major global health burden, necessitating innovative preventive approaches. *Artemisia annua* (*A. annua*), known for its extensive pharmacological properties, has shown potential in cancer therapy. This study investigates the chemopreventive efficacy of methanolic extract of *A. annua* (MEA) in an azoxymethane (AOM)-induced murine model of CRC, with a focus on its antioxidant, biomarker modulation, and pro-apoptotic activities. **Methods:** MEA was obtained via cold solvent extraction, yielding 39%, and demonstrated potent in vitro cytotoxicity against HCT116 and RKO colon cancer cell lines, with IC50 values of 20 µg/mL and 15 µg/mL, respectively. Swiss albino mice were treated with MEA beginning two weeks before AOM induction, with treatment continuing for 21 weeks. Survival was monitored for 40 weeks. Key outcomes included serum biomarker levels (ADA, GGT, CD73, LDH), antioxidant enzyme activities (SOD, CAT, GPx1, MDA), reactive oxygen species (ROS) modulation, apoptosis induction, and histopathological evaluation. **Results:** MEA significantly improved survival rates, reduced AOM-induced weight loss, and modulated cancer biomarkers, with marked reductions in ADA, GGT, CD73, and LDH levels. Antioxidant defenses were restored, as evidenced by increased SOD, CAT, and GPx1 activities and decreased MDA levels. ROS levels were significantly reduced, and apoptosis in colonic cells was effectively induced. Histopathological analysis revealed substantial mitigation of CRC-associated morphological abnormalities. **Conclusions:** MEA exhibits robust chemopreventive properties, demonstrating its potential to reduce oxidative stress, modulate key biomarkers, and induce apoptosis in CRC. These findings position MEA as a promising natural candidate for CRC prevention and therapy, warranting further exploration for clinical application.

## 1. Introduction

According to GLOBOCAN 2022, cancer continues to be one of the major causes of mortality around the globe, accounting for nearly 10 million deaths each year. The prevalent forms of cancer encompass lung, breast, colorectal, and prostate malignancies. Efforts aimed at prevention, early detection, and enhanced treatments are paramount in addressing this global health challenge. Colorectal cancer (CRC) is a substantial global health concern, affecting millions of individuals annually, with an estimated 1.4 million new cases reported worldwide each year. Despite the significant advancements in the field of medical science, colorectal cancer (CRC) continues to be a prominent cause of mortality worldwide [1,2,3,4]. In the Kingdom of Saudi Arabia, there has been a consistent increase in the incidence of CRC. Recent estimates reveal that CRC is now the second most prevalent cancer among males and the third most prevalent among females in the country [1,5,6]. Conventional treatment modalities, including surgery, chemotherapy, and radiation therapy, have demonstrated encouraging outcomes. Nevertheless, there are ongoing challenges in the form of drug resistance and the occurrence of toxicities associated with these treatments [7,8,9]. As a result, researchers and clinicians are actively seeking alternative approaches that have the potential to improve patient outcomes and address the increasing public health challenge.

In recent years, there has been a surge of interest in the use of herbal medicines for cancer treatment and chemoprevention. These natural compounds, many of which have been used for centuries, may be effective against cancer either alone or in combination with existing chemotherapeutic drugs [10,11]. Among them, extracts derived from black seeds that contain a high concentration of thymoquinone have demonstrated encouraging outcomes in the stimulation of apoptosis and the inhibition of cancer cell proliferation [10,11,12,13,14,15,16,17,18,19,20]. Tea polyphenols, specifically epigallocatechin gallate, demonstrate antiangiogenic and proapoptotic properties in different types of cancer [17,18]. Curcumin, which is obtained from turmeric, exhibits diverse mechanisms for inhibiting cancer by modulating various signaling pathways [19,20]. The potential of fenugreek seed extracts and gingerol in suppressing tumor progression and metastasis is significant [21,22,23,24,25,26]. Resveratrol, derived from the skin of red grapes, demonstrates chemopreventive and antiproliferative effects on cancer cells [27,28,29].

*Artemisia annua* (known as sweet wormwood) has long been valued in traditional medicine for its diverse therapeutic properties attributed to a rich array of phytochemicals. Among its bioactive compounds, artemisinin—a sesquiterpene lactone—has gained prominence for its potent antimalarial activity, revolutionizing malaria treatment and earning a Nobel Prize in Physiology or Medicine in 2015 [30,31,32,33]. Beyond its antimalarial properties, artemisinin has also demonstrated anticancer activity, along with other bioactive compounds in *A. annua*, such as sesquiterpenes, polyphenols, and flavonoids. Together, these phytochemicals contribute to the plant’s broader pharmacological profile, which includes antioxidant, anti-inflammatory, and cytotoxic activities [34,35,36,37,38].

Emerging preclinical and clinical studies highlight the cytotoxic effects of *A. annua* against multiple cancer cell types, underscoring their potential as promising candidates in cancer research. The antitumor activity of bioactive constituents of *A. annua* is multifaceted, involving the induction of apoptosis, inhibition of cell proliferation, disruption of angiogenesis, and targeting of cancer stem cells. Furthermore, it synergizes with conventional chemotherapeutic agents, such as 5-fluorouracil (5-FU) and doxorubicin, enhancing treatment efficacy and reducing drug resistance. These findings have been validated across various cancer types, including breast, lung, colon, liver, leukemia, and melanoma [39,40,41,42,43,44,45,46,47]. In colorectal cancer (CRC), it exerts its therapeutic effects through mechanisms such as downregulating pro-inflammatory cytokines, suppressing nuclear factor-kappa B (NF-κB) activity—a key regulator of inflammation and tumorigenesis—and inhibiting the Wnt/β-catenin signaling pathway, a crucial driver of CRC progression and metastasis [48,49,50,51,52]. By synergizing with standard chemotherapies, artemisinin not only enhances therapeutic outcomes but also allows for reduced drug dosages, thereby minimizing associated toxicities [53,54].

The growing interest in using *A. annua* and artemisinin to treat infectious diseases and cancer highlights the need for additional research to comprehend their pharmacological actions and fully maximize their therapeutic potential. The present study focuses on assessing the chemopreventive efficacy of MEA in a mouse model of AOM-induced CRC in Swiss albino mice. It also attempted to determine the potential molecular targets of MEA against CRC in vivo.

## 2. Results

### 2.1. Yield Percentage of MEA and GC-MS Analysis

**Figure 1:**
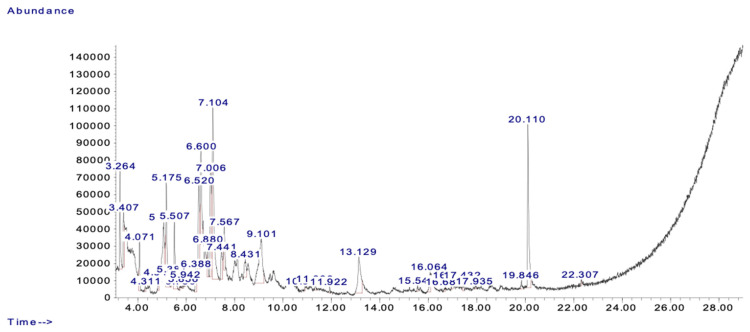
Chromatogram of bioactive compounds identified in the methanolic extract of *Artemisia annua* (MEA).

The yield percentage of MEA was calculated as 39% after the dried extract weighed in at 39 g. The GC-MS analysis of MEA identified 18 compounds, including nonadecane, 3-pyrrolidinol, quinoline derivatives, and benzenepropanoic acid derivatives (Figure 1, Table 1). These compounds have been associated with biological activities, such as cytotoxicity, antioxidant effects, and anti-inflammatory properties, which align with MEA’s observed chemopreventive effects [55,56,57,58]. While toxicity was not evaluated in this study, future investigations should focus on exploring the safety and dose-dependent effects of MEA to ensure their therapeutic applicability.

### 2.2. Evaluation of MEA’s Anticancer Activity in HCT116 and RKO Colon Cancer Cell Lines

**Figure 2:**
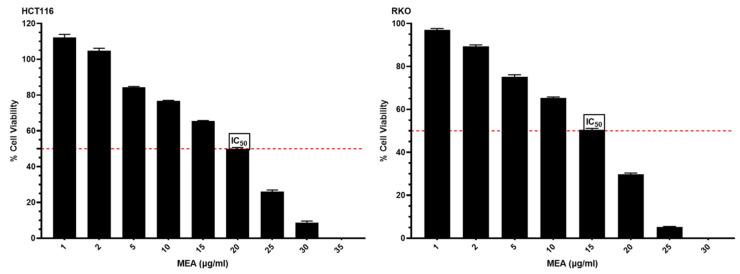
Assessment of MEA’s concentration-dependent impact on cell proliferation. This figure illustrates the determination of the half-maximal inhibitory concentration (IC_50_) of MEA on the proliferation of HCT116 and RKO colon cancer cell lines. The evaluation was conducted using a cell cytotoxicity assay over 48 h, with varying concentrations of MEA.

The findings of the cell cytotoxicity tests showed that both colon cancer cell lines were very sensitive to MEA. According to the findings, the IC_50_ of MEA was determined to be 20 μg/mL in HCT116 cells and 15 μg/mL in RKO colon cancer cells (Figure 2). These results highlight the promising in vitro anticancer properties of MEA against colon cancer.

### 2.3. Effects of MEA on AOM-Induced ABW and Survival

**Figure 3:**
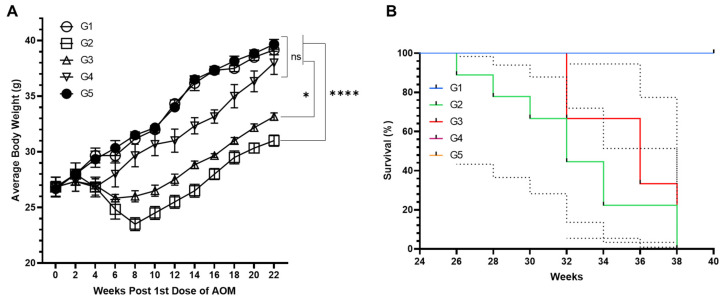
Analysis of MEA’s influence on carcinogen-induced effects. This figure illustrates (**A**) the changes in average body weight (ABW) and (**B**) the survival rates under the influence of MEA. Part (**A**) data represent the standard error of the mean (SEM) from a sample of five animals per group. For part (**B**), the survival data are based on a sample size of ten mice per group. ’ns’ denotes no significant difference. The symbol ‘*’ signifies a statistically significant difference between groups at a *p*-value less than 0.05, while ‘****’ represents a more pronounced statistical significance, indicating a *p*-value of less than 0.0001.

**Figure 4:**
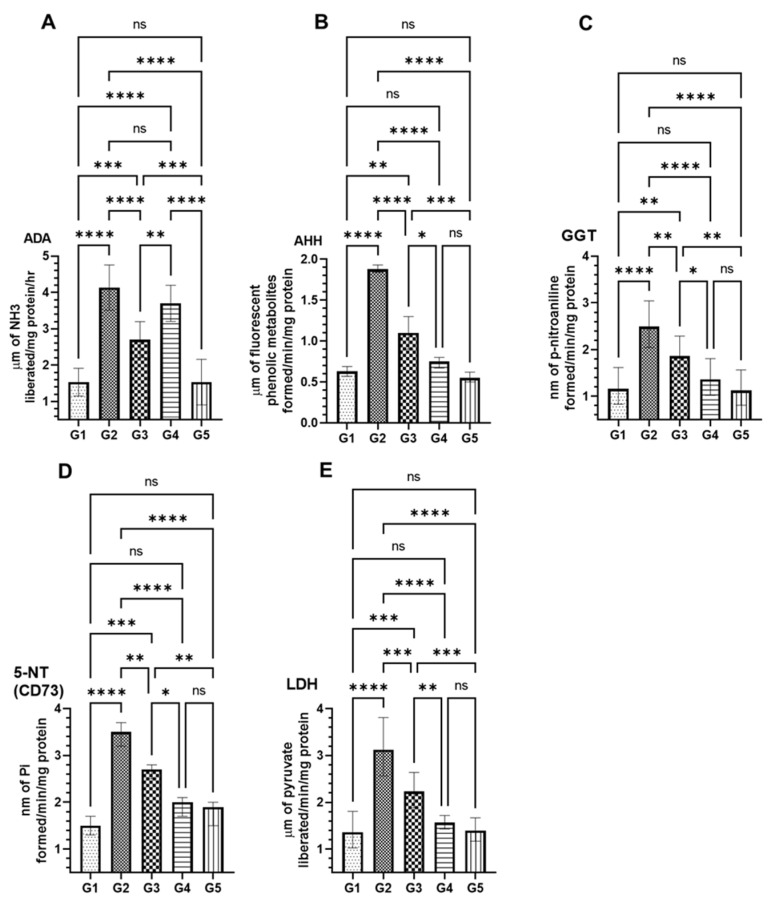
Analysis of MEA’s impact on serum levels of AOM-induced cancer marker enzymes. This figure presents the effects of MEA on (**A**) adenosine deaminase (ADA), (**B**) aryl hydrocarbon hydroxylase (AHH), (**C**) gamma-glutamyl transferase (GGT), (**D**) 5′-nucleotidase (5-NT/CD73), and (**E**) lactate dehydrogenase (LDH) in serum. Data are expressed as the SEM derived from three independent experiments. ’ns’ indicates no significant difference within the treated groups. Symbols ‘*’, ‘**’, ‘***’, and ‘****’ denote statistically significant differences between groups with *p*-values less than 0.05, 0.01, 0.001, and 0.0001, respectively.

The data revealed a notable disparity in the mice’s average body weights (ABWs) subjected to experimental conditions. In particular, G2, which was exposed to AOM, exhibited a significantly lower ABW of 31.0 g at the 22-week mark before euthanasia when compared to the 39.16 g observed in both the control group (G1) and group 5 (G5). A distinct reduction in ABW was recorded between the 2nd and 8th weeks, with weights declining from 28.0 g to 23.5 g, as depicted in Figure 3A. Subsequently, there was an increase in ABW within G2. However, it failed to return to the baseline levels seen in G1 or the values recorded for group 4 (G4), also illustrated in Figure 4A. Further analysis indicated that the ABW of the mice in group 3 (G3), which received a low dose of MEA, diminished significantly. In contrast, the ABW of mice in G4, pre-treated with a high dose of MEA for the same 22-week duration, remained stable, showing no significant fluctuations from the baseline established by G1/G5. An exception was noted during the 4th week within G4, which was exposed to AOM; a transient decrease in ABW was observed in group 4 (G4), followed by a consistent upsurge in the subsequent weeks.

Extending the observation period to 40 weeks, the Kaplan–Meier survival analysis presented a divergent mortality landscape across the groups. Specifically, G3, which received a low dose of MEA, exhibited a mortality rate of 40%. In stark contrast, mice in G4 that received a high dose of MEA demonstrated a 100% survival rate. Meanwhile, a significant mortality rate of 80% was noted in G2, the group solely exposed to AOM, as shown in Figure 3B.

### 2.4. Influence of MEA on Serum Cancer Marker Enzymes in AOM-Exposed Mice

The results revealed significant alterations in serum cancer marker enzyme levels following treatment with Methanol Artemisia Extract (MEA) in groups G3 and G4. These changes were in response to the enzymatic modifications induced by AOM in the G2 group, as benchmarked against the vehicle-treated control (G1) and the MEA-only group (G5). As illustrated in Figure 4, exposure to AOM resulted in elevated levels of several enzymes: adenosine deaminase (ADA) increased to 4.13 µm ± 0.14 SEM, gamma-glutamyl transferase (GGT) to 2.5 µm ± 0.11 SEM, ecto-5′-nucleotidase (CD73) to 3.46 µm ± 0.15 SEM, and lactate dehydrogenase (LDH) to 3.13 µm ± 0.14 SEM. In comparison, the levels of these enzymes in the serum of the vehicle-treated control group (G1) were ADA at 1.53 µm ± 0.09 SEM, AHH at 0.63 µm ± 0.04 SEM, GGT at 1.16 µm ± 0.09 SEM, CD73 at 1.5 µm ± 0.11 SEM, and LDH at 1.36 µm ± 0.09 SEM. A significant reduction in the levels of these markers was noted in group G3, which received a lower MEA dose, and more markedly in group G4 with a higher MEA dose: ADA levels decreased to 2.7 µm ± 0.11 SEM in G3 and to 3.7 µm ± 0.11 SEM in G4; GGT to 1.86 µm ± 0.08 SEM in G3 and to 1.37 µm ± 0.09 SEM in G4; CD73 to 2.66 µm ± 0.09 SEM in G3 and to 1.93 µm ± 0.15 SEM in G4; and LDH to 2.23 µm ± 0.09 SEM in G3 and to 1.56 µm ± 0.03 SEM in G4. Additionally, a significant decrease in aryl hydrocarbon hydroxylase (AHH) was observed in G2 at 1.88 µm ± 0.03 SEM compared to G1 at 0.63 µm ± 0.04 SEM, which was notably ameliorated in the MEA-treated groups (1.1 µm ± 0.11 SEM in G3 and 0.74 µm ± 0.37 SEM in G4). It is important to note that group G5, treated solely with MEA, showed no significant changes in enzyme activity compared to the G1 control group (Figure 4).

### 2.5. Modulation of Antioxidant Enzymes by MEA in AOM-Exposed Colonic Tissues

**Figure 5:**
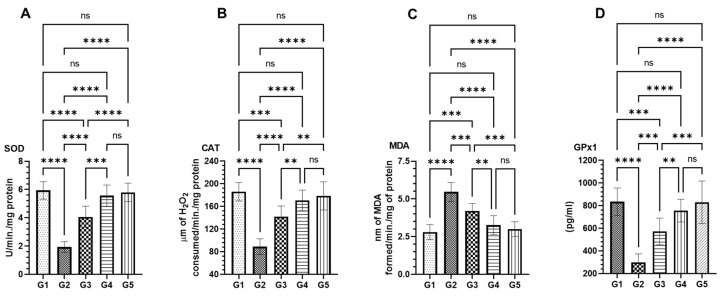
Assessment of MEA’s influence on antioxidant enzymes in colon tissue. This figure depicts the effects of MEA on various antioxidant enzymes, including (**A**) superoxide dismutase (SOD), (**B**) catalase (CAT), (**C**) malondialdehyde (MDA), and (**D**) glutathione peroxidase 1 (GPx1). Data are expressed as the SEM based on results from three separate experiments. ‘ns’ denotes no significant differences within the groups. Symbols ‘**’, ‘***’, and ‘****’ indicate statistically significant differences within the groups, corresponding to *p*-values of less than 0.05, 0.01, 0.001, and 0.0001, respectively.

Analysis of antioxidant enzyme activities revealed a notable restoration of superoxide dismutase (SOD), catalase (CAT), malondialdehyde (MDA), and glutathione peroxidase (GPx1) levels in the groups pre-treated with MEA, specifically in groups G3 and G4. This restoration appeared to counteract the alterations induced by AOM exposure in group G2 (Figure 5). Quantitatively, the SOD activity was elevated to 4.06 U ± 0.17 SEM in the G3 group treated with a lower dose of MEA and to 5.56 ± 0.15 SEM in the G4 group treated with a higher dose. This increase was significant compared to the 1.93 U ± 0.09 SEM observed in the AOM-exposed G2 group (Figure 5A). For CAT activity, the G3 group showed a level of 142 µm ± 4.36 SEM, and the G4 group had a level of 170 µm ± 5.78 SEM, both markedly higher than the 89.0 µm ± 5.56 SEM measured in the G2 group (Figure 5B). MDA levels, indicative of lipid peroxidation, were substantially reduced in the MEA pre-treated groups. The G2 group showed an increased level at 5.46 nm ± 0.15 SEM following AOM exposure, compared to 2.8 nm ± 0.12 SEM in the control G1 group. This elevation was mitigated in the G3 group to 4.2 nm ± 0.11 SEM and further to 3.26 nm ± 0.14 SEM in the G4 group (Figure 5C). Finally, GPx1 levels exhibited a significant rebound from the decrease induced by AOM in G2 (300 pg ± 17.32 SEM) to 573.3 pg ± 27.28 SEM in G3 and 756.7 pg ± 23.33 SEM in G4, both improvements over the baseline level of 835 pg ± 28.43 SEM seen in the untreated G1 group (Figure 5D). It is also noteworthy that the MEA-only treated G5 group did not display alterations in these enzymes compared to the vehicle-treated control G1 group (Figure 5).

### 2.6. Assessment of Reactive Oxygen Species in Colonic Cells by DCFDA Flow Cytometry

**Figure 6:**
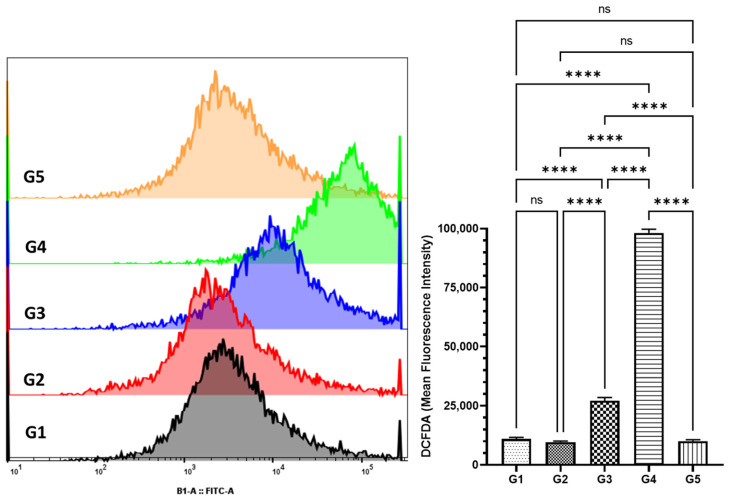
Evaluation of MEA’s impact on cellular reactive oxygen species (ROS) in colon cells. This figure demonstrates the effects of MEA on cellular ROS levels, as measured by 2′,7′-dichlorofluorescein diacetate (DCFDA) assay and analyzed using flow cytometry. Data are presented as the standard error of the mean (SEM) based on three independent experiments. ‘ns’ indicates no significant difference within the groups. The ‘****’ symbol represents a statistically significant difference between the groups, with a *p*-value of less than 0.0001.

The analysis of ROS levels in colon cells was performed using DCFDA staining. The mean fluorescence intensity (MFI) was quantified using FlowJo software v10.8.1 after data acquisition with the MACSQuant Analyzer. The findings indicated a pronounced increase in cellular ROS in the azoxymethane (AOM)-exposed group, G2, with an MFI of 9667.1 ± 422.65 SEM. This level was similar to the control group, G1, and the MEA-only group, G5, which had MFIs of 10,992.42 ± 679.1 SEM and 10,100 ± 568.5 SEM, respectively (Figure 6). Interestingly, Methanol Artemisia Extract (MEA) demonstrated a chemopreventive effect by significantly diminishing the levels of cellular ROS. In the groups pre-treated with MEA, G3 and G4, the recorded MFIs were 27,200 ± 1300 SEM and 98,176.67 ± 750.025 SEM, respectively, indicating a substantial reduction in ROS levels (Figure 6).

### 2.7. Assessment of Apoptosis in Colonic Cells by Annexin V-FITC-PI Flow Cytometry

**Figure 7:**
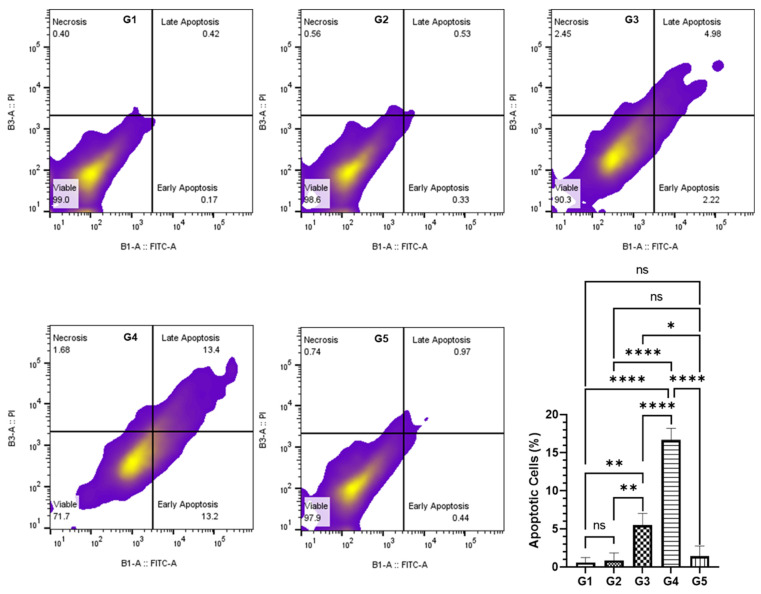
Analysis of MEA’s effect on apoptosis induction in colon cells. This figure illustrates the impact of MEA on apoptosis in colon cells, as determined by annexin V-FITC and propidium iodide (PI) staining, with results analyzed using flow cytometry. Data are represented as the standard error of the mean (SEM) compiled from three experiments. ‘ns’ signifies no significant difference within the groups. The symbols ‘*’, ‘**’, and ‘****’ denote statistically significant differences between groups, with a *p*-value of less than 0.05, 0.01, and 0.0001, respectively.

The evaluation of apoptotic cell populations within the colonic epithelium was conducted through dual staining with annexin V-FITC and propidium iodide (PI). This staining discriminates live cells, early apoptotic, late apoptotic, and necrotic cells. Post-staining, the cell samples were subjected to flow cytometric analysis employing the MACSQuant Analyzer. Subsequent data interpretation was facilitated by FlowJo software. The quantitative analysis revealed that in G4, consisting of mice that received a higher dosage of MEA with AOM exposure, there was a substantial elevation in apoptotic cell frequency, amounting to 16.67% of the total cell population. Conversely, in G3, which received a lower dose of MEA, the proportion of apoptotic cells was notably less, recorded at 5.5%. These observations underscore the potentiation of apoptosis by MEA in cells that sustained damage through AOM exposure, which presumably failed to undergo a DNA repair mechanism. Conversely, the other experimental groups did not demonstrate any appreciable elevation in apoptotic levels, as evidenced in Figure 7.

### 2.8. Assessment of Colon Tissues by H&E Staining

**Figure 8:**
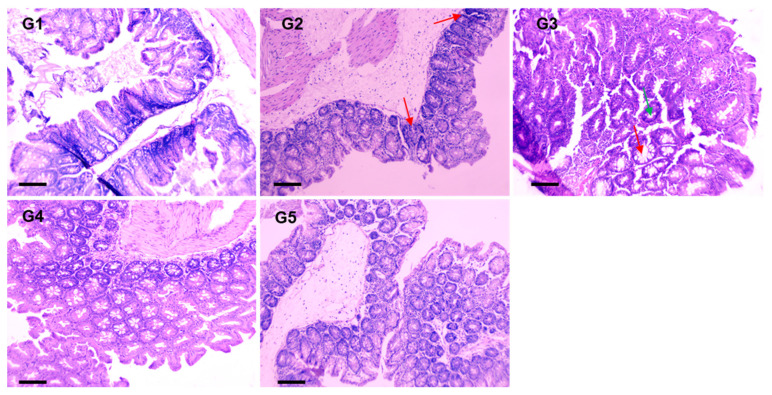
Histopathological analysis of MEA’s effect on AOM-induced colorectal carcinogenesis. Representative histopathological images of colon tissue showing the effects of MEA on AOM-induced carcinogenesis. Images are displayed at 100× magnification, with a scale bar indicating 100 µm.

The histopathological examination using Hematoxylin and Eosin (H&E) staining revealed morphological changes associated with colon cancer development. In the AOM-treated group (G2), significant histological aberrations were observed, including irregularities in the size and shape of mucosal glands, infiltration of acinar glands into the muscularis mucosae (marked by red arrows), and prominent vascular congestion. In contrast, the groups pre-treated with MEA (G3 and G4) demonstrated notable histological improvements, underscoring MEA’s potential to inhibit tumor progression. These groups’ submucosal and muscular layers appeared essentially normal, with minimal hyperchromatic cells observed (indicated by green arrows). However, some swelling in the mucosal glands was noted in G3 (marked by red arrows), whereas in G4, these glands exhibited more regular and near-normal morphology. The group receiving only MEA (G5), which served as a negative control, preserved the normal histological architecture, characterized by typical mucosal gland structures, intact submucosal and muscular layers and no pathological alterations attributable to MEA treatment alone (Figure 8).

## 3. Methods

### 3.1. Materials

Azoxymethane (AOM) was purchased from Sigma-Aldrich in St. Louis, Missouri, USA. The annexin V-FITC apoptosis assay kit, crucial for our apoptosis detection studies, was obtained from Miltenyi Biotec, Bergisch Gladbach, Germany, known for their precision in biotechnological reagents. From Abcam, Cambridge, MA, USA, we procured the 2′,7′-dichlorofluorescein diacetate (DCFDA), alongside cancer marker and antioxidant enzyme assay kits, selected for their proven efficacy in similar research contexts. The dried leaves of *A. annua*, integral to our study, were sourced from a certified local pharmacy in Buraydah, Saudi Arabia, ensuring authenticity and local relevance. All other chemicals and laboratory supplies were acquired from regional vendors and chosen for their quality assurance and proximity, facilitating consistent supply throughout the study.

### 3.2. Methanolic Extraction of A. annua (MEA) Using Cold Maceration Technique

The cold solvent extraction method was chosen to preserve heat-sensitive bioactive compounds, while methanol was employed for its efficiency in extracting a wide range of phytochemicals. The extraction protocol was adapted from a previously established method, with slight modifications [47,59]. To prepare the methanolic extract of *A. annua* (MEA), dried leaves were ground into a coarse powder (100 g) and defatted using cyclohexane. The powder was placed in a beaker with 300 mL of cyclohexane and stirred continuously for 4 h. The mixture was centrifuged at 5000 rpm for 10 min, and the supernatant containing cyclohexane, lipids, and other soluble impurities was discarded. The defatted material was air-dried at 40 °C overnight. The dried, defatted powder was transferred to a fresh beaker for methanolic extraction, and 300 mL of pure methanol was added. The mixture was stirred at ambient temperature for 72 h to ensure comprehensive extraction of bioactive compounds. The extract was then filtered using Whatman No. 1 filter paper to remove solid residues. The filtrate was concentrated by evaporating the solvent under a nitrogen atmosphere using a rotary evaporator. This process yielded the crude methanolic extract of *A. annua* in a dry, powdered form.

### 3.3. GC-MS (Gas Chromatography-Mass Spectrometry) Analysis

The analysis of bioactive compounds in the methanolic extract of *A. annua* (MEA) was performed using a Gas Chromatography-Mass Spectrometry (GC-MS) system consisting of an Agilent GC 7890A coupled with a 5975C triple-axis detector single-quadrupole mass spectrometer. An Agilent HP-5MS capillary column (30 m × 0.25 mm × 0.25 µm film thickness) was employed for compound separation. High-purity helium served as the carrier gas at a constant 1 mL/min flow rate.

The injector temperature was maintained at 230 °C, utilizing a splitless injection mode with a split ratio of 20:1. The column temperature program began at 40 °C, held for 1 min, followed by a linear ramp to 150 °C at a rate of 10 °C/min (held for 2 min). The temperature was further increased to 300 °C at 10 °C/min, with a final hold of 1 min. The mass spectrometer operated at an ion source temperature of 150 °C, with an interface line temperature set to 280 °C. Electron ionization (EI) was conducted at 70 eV, scanning a mass range from 50 to 550 *m*/*z*. A solvent delay of 3 min was applied to prevent interference from the solvent peak.

Compounds were identified by matching the obtained spectra with entries in the National Institute of Standards and Technology (NIST) 2008 library. Each analysis required approximately 40 min per sample. This approach enabled the precise characterization of the bioactive components present in MEA.

### 3.4. In Vitro Cell Cytotoxicity Assay

The primary screening of anticancer activity of MEA was conducted by measuring the viability percentage of HCT116 and RKO colon cancer cells at different concentrations using a cell cytotoxicity assay kit from Abcam. Briefly, the cells were seeded into 96-well cell culture plates at a 70–80% exponential confluency density, with 10,000 cells per well. The cells were allowed to incubate for 24 h in the incubator at 37 °C with 5% CO_2_. Then, the cells were treated to different concentrations of MEA ranging from 0.1 μg/mL to 100 μg/mL and incubated for 48 h. Per the manufacturer’s instructions, the cell cytotoxicity reagent (20l) was added to each well after treatment. Subsequently, the microplate was incubated at 37 °C, and the measurements were taken at a wavelength of 590 nm using a microplate reader. The measurement of cell viability was conducted using the following formula:

$$\%\;Cell\;Viability=100\times \frac{\left(A_{sample}-A_{0}\right)}{\left(A_{Ctrl}-A_{0}\right)}$$

*A_sample_* represents the absorbance from cells treated with MEA.

*A_ctrl_* denotes the absorbance obtained from the cells that did not receive any treatment.

*A*_0_ corresponds to the background absorbance level, which is determined using a control consisting only of media without any cells.

### 3.5. In Vivo Studies

#### 3.5.1. Mice

In this study, female Swiss albino mice aged 8 to 10 weeks were used as the experimental subjects. These animals were sourced from the animal research facility at King Saud University, Riyadh, Saudi Arabia. All in vivo procedures adhered to the ethical guidelines set by the Animal Welfare Society, University of London, Wheathampstead, UK. The study aimed to induce colorectal cancer (CRC) in mice using azoxymethane (AOM) as a carcinogen. In addition to the induction of CRC, the experimental procedures involved blood collection, injection administration, and the humane euthanasia of the mice.

The research protocol, 24-01-01, was approved by the Animal Ethical Committee of Qassim University, Saudi Arabia. The mice were housed in the CAMS animal facility, where their care followed strict compliance with institutional animal welfare guidelines. During the experiment, trained staff performed health checks on the animals twice daily to ensure their well-being. At the end of this study, the mice were euthanized by approved ethical protocols, utilizing CO_2_ inhalation, a method considered humane and appropriate for euthanasia. This process was carried out over 2–4 h and applied to all animals, including those in a moribund state or exhibiting reduced responsiveness. Notably, no deaths occurred before the scheduled euthanasia, ensuring that all experimental mice were accounted for throughout the study.

#### 3.5.2. Experimental Design

**Figure 9:**
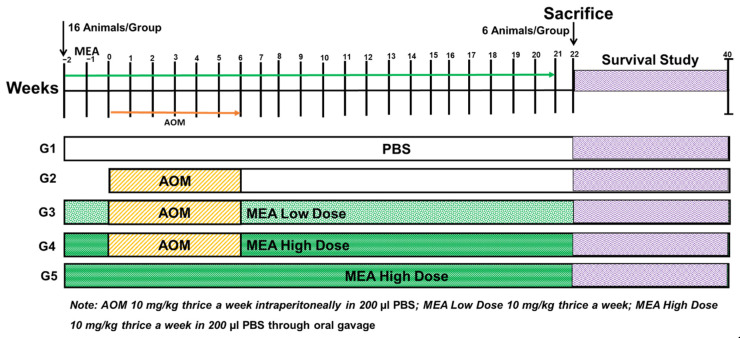
In vivo study design overview. Group 1 (control): Administered Phosphate-Buffered Saline (PBS) orally thrice weekly from week −2 to week 21. Group 2: Received azoxymethane (AOM) at a dose of 10 mg/kg body weight in 200 µL PBS, administered intraperitoneally three times weekly from week 0 to week 6. Group 3 (MEA Low Dose): Treated with MEA at 10 mg/kg in PBS following the schedule of Group 1, combined with AOM treatment as in Group 2. Group 4 (MEA High Dose): Received MEA at 20 mg/kg in PBS, with the Group 1 + AOM regimen as in Group 2. Group 5: Treated with a high dose of MEA (20 mg/kg) following the Group 1 protocol. PBS and MEA were given orally to all groups, while AOM was administered intraperitoneally.

A total of 80 mice were randomly assigned to five groups, each containing 16 animals. As shown in Figure 9, the intraperitoneal (IP) administration of 10 mg/kg b.w of AOM in 200 µL of PBS three times a week for six weeks was used to initiate and promote the CRC. The administration of MEA (10 or 20 mg/kg b.w) was initiated through oral gavage, starting 2 weeks before the initial AOM dosage, and was maintained for 21 weeks, as described earlier [55]. Six animals from each group were chosen for sacrifice 22 weeks after the initial treatment of AOM to perform further biochemical and histopathological investigations, as described previously [14,21,47,60]. If the mice exhibited a lack of response, they were humanely euthanized within 24 h according to the protocols outlined in the observational study, and these instances were noted in the survival analysis data.

### 3.6. Study of Average Body Weight (ABW) and Survival Rate

Each group’s average body weight (ABW) was recorded at week 0 and subsequently monitored at 2-week intervals for 22 weeks. Following the initial AOM exposure, all surviving animals were followed for another 18 weeks until the end of week 40.

### 3.7. Histopathological Evaluation of Colon Tissues

Histopathological analysis of colon tissues was conducted to evaluate structural alterations induced by AOM and the protective effects of MEA. Formalin-fixed tissues were embedded in paraffin, sectioned, and stained with Hematoxylin and Eosin (H&E). For each sample, 100 random fields were analyzed under a light microscope at 100× magnification to ensure a comprehensive evaluation and minimize sampling bias. Morphological changes such as glandular irregularities, infiltration of acinar glands, vascular congestion, and preservation of normal histological architecture were assessed. Representative images of the histological findings are provided in the manuscript.

### 3.8. The Effect of MEA on the Levels of Cancer Marker Enzymes in the Serum Induced by AOM

This study examined the efficacy of different formulations in the serum by assessing the activities of carcinogenesis markers, including ADA, LDH, γ-GT, and 5′-NT. Abcam kits were utilized according to the instructions provided for each kit.

### 3.9. The Effect of MEA on Antioxidant Enzymes in the Colon Induced by AOM

To assess the potential of MEA on antioxidant enzymes, the enzymatic activities of superoxide dismutase (SOD), catalase (CAT), malondialdehyde (MDA), and glutathione peroxidase 1 (GPx1) were analyzed in the colonic tissues of the treated animal groups. In a summarized procedure, the excised colonic tissues were subjected to centrifugation using the buffers provided in the respective enzyme assay kits, strictly following the guidelines recommended by the kit manufacturers.

### 3.10. Annexin V-FITC/PI Apoptotic Assay

The cell distribution in colon tissue was examined using the MACSQuant Analyzer, focusing on differentiating cellular states based on annexin V-FITC and PI staining. Briefly, the process began with the preparation of a single-cell suspension from the colonic tissues of each experimental group. Subsequently, the cells were filtered through a cell strainer featuring a 100 µm mesh and then centrifuged at 30× *g*. The resultant cell pellet was resuspended in a specific binding buffer. The next step involved staining the cells with annexin V-FITC-PI, as provided in the kit, for 20–25 min. Post-staining, the cells were processed in the MACSQuant Analyzer 10 (Miltenyi Biotec, Bergisch Gladbach, Germany). The analysis of the cell distributions was conducted using FlowJo software version 10.8.1, which facilitated the generation of cell distribution plots for each sample.

### 3.11. Assessment of Reactive Oxygen Species (ROS) in Colonic Cells Using Flow Cytometry

Following the sample acquisition in the MACSQuant Analyzer, DCFDA staining of the colonic cells was measured as the mean fluorescence intensity (MFI) using FlowJo. After being filtered through a cell strainer with a 100 μm mesh cell strainer and centrifuged at 300× *g*, the cells were suspended in the DMEM medium. The cells were then stained with a 20 µM concentration of DCFDA and incubated for 40 min at 37 °C. The samples were processed post-incubation using the MACSQuant Analyzer 10. The final analysis involved calculating the MFI of DCFDA for each sample, with histograms generated using FlowJo software v10.8.1.

## 4. Discussions

This study demonstrates the significant chemopreventive and anticancer potential of the methanolic extract of *A. annua* (MEA) against colorectal cancer (CRC). MEA showed potent cytotoxic activity in vitro against HCT116 and RKO colon cancer cell lines, with IC50 values of 20 µg/mL and 15 µg/mL, respectively (Figure 3). These findings align with previous research reporting the anticancer properties of *A. annua* extracts, which have been shown to induce apoptosis and arrest the cell cycle in various cancer models [30,61,62]. Furthermore, the efficient extraction of MEA, yielding 39%, highlights the success of the cold solvent extraction method in isolating bioactive constituents from *A. annua* [63,64].

While this study primarily focused on MEA’s chemopreventive effects, its selective cytotoxicity on normal cells versus cancer cells remains unexplored. The in vitro experiments provided insights into its cytotoxic potential, but future research should assess its impact on normal cells to better understand its therapeutic index. A preliminary acute toxicity assay in mice indicated no observable adverse effects at the tested doses (data not included), indicating MEA’s safety. However, further studies are warranted to comprehensively evaluate its toxicity and therapeutic potential.

The in vivo assessment of MEA in the azoxymethane (AOM)-induced CRC model provided robust evidence of its protective effects. AOM exposure significantly reduced average body weight (ABW) in the untreated group (G2), a standard marker of tumor burden and disease progression [60,65]. Treatment with a higher dose of MEA (G4) effectively stabilized ABW, indicating its ability to counteract cancer-associated weight loss. As previously reported by Kim et al., this protective effect may stem from the antioxidant and anti-inflammatory properties of *A. annua* [66]. Furthermore, the Kaplan–Meier survival analysis revealed a marked survival benefit in the G4 group, with a 100% survival rate compared to 80% mortality in the AOM group (G2). These results suggest that MEA not only inhibits tumor progression but may also enhance overall health and resilience to cancer-related stress.

A dose-dependent relationship was observed, with the low-dose group (G3) showing a 40% mortality rate, whereas the high-dose group (G4) achieved complete survival. This highlights the importance of determining optimal dosages to maximize therapeutic efficacy while minimizing potential toxicity. Similar dose-dependent outcomes have been observed in studies on other plant extracts, where the therapeutic response correlates closely with the administered dosage [14,21,60,67].

MEA demonstrated significant regulation of biochemical markers associated with cancer progression. Elevated levels of adenosine deaminase (ADA), gamma-glutamyl transferase (GGT), ecto-5′-nucleotidase (CD73), and lactate dehydrogenase (LDH) in the AOM-treated group (G2) were substantially reduced by MEA treatment, particularly in the high-dose group (G4). These findings align with previous studies indicating the role of these enzymes in carcinogenesis and tumor growth [68,69]. Additionally, the reduction in aryl hydrocarbon hydroxylase (AHH) activity by MEA suggests its potential role in modulating carcinogen metabolism and mitigating DNA damage induced by carcinogens [70].

MEA’s impact on oxidative stress and antioxidant defense was also noteworthy. The extract restored the activity of key antioxidant enzymes, including superoxide dismutase (SOD), catalase (CAT), and glutathione peroxidase (GPx1), while reducing malondialdehyde (MDA) levels in colonic tissues. The elevated SOD and CAT activity in the MEA-treated groups suggests enhanced neutralization of reactive oxygen species, consistent with findings from other plant-derived antioxidants [67,68,69,70]. The decrease in MDA levels further underscores MEA’s role in mitigating oxidative damage, a critical factor in cancer progression [14,21,47,60,67,71].

Flow cytometry analysis using annexin V-FITC and PI staining revealed a dose-dependent increase in apoptosis in the high-dose MEA group (G4), confirming MEA’s ability to induce apoptotic cell death in AOM-exposed cells. Apoptosis induction is crucial for eliminating potentially malignant cells, reinforcing MEA’s chemopreventive efficacy. The histopathological analysis corroborated these findings, demonstrating that MEA preserved the normal colonic tissue architecture and prevented the dysplastic changes commonly associated with colorectal carcinogenesis.

These findings underscore MEA s potential as a chemopreventive agent through its regulation of key biomarkers, enhancement of antioxidant defenses, and induction of apoptosis. MEA’s ability to stabilize body weight, improve survival, and modulate cancer-related biochemical markers highlights its therapeutic promise. Future research should aim to elucidate the molecular mechanisms underlying these effects and further validate MEA’s role as a complementary strategy in cancer prevention.

## 5. Conclusions

This study provides strong evidence of the chemopreventive and anticancer potential of the methanolic extract of *Artemisia annua* (MEA) in colorectal cancer (CRC) models. MEA exhibited significant cytotoxicity against colon cancer cell lines and demonstrated protective effects in azoxymethane (AOM)-induced CRC in mice, including body weight stabilization and improved survival rates. MEA also modulated key serum cancer marker enzymes, restored antioxidant enzyme levels, and reduced oxidative stress, highlighting its role in mitigating carcinogenesis. The dose-dependent increase in apoptosis and preserving normal tissue architecture further underscores its therapeutic potential. These findings lay a foundation for further exploration of the molecular mechanisms underlying MEA’s effects and support its development as a promising complementary agent in CRC prevention and management.

## Figures and Tables

**Table 1:** Biologically active chemical compounds of methanolic extracts of *Artemisia annua* (MEA).

Compound Number (#)	Retention Time RT (min)	Area (Ab*s)	Baseline Heigth (Ab)	Absolute Heigth (Ab)	Peak Width 50% (min)	Hit Number	Hit Name	Probability Ratio	Mol Weight (amu)
**1**	3.407	83,860	31,696	49,692	0.063	1	Toluene	76	92.063
**2**	5.507	138,288	39,649	44,873	0.177	1	Benzene, 1,3-dimethyl-	97	106.078
**3**	5.736	4864	2405	6815	0.046	1	2,4-Octadiyne	38	106.078
**4**	5.856	4569	2542	8112	0.057	1	3-Pyrrolidinol	9	87.068
**5**	5.942	7651	3902	10,694	0.051	1	Benzene, (1-methylethyl)-	52	120.094
**6**	6.388	43,149	13,318	16,754	0.086	1	Benzene, propyl-	87	120.094
**7**	6.52	215,496	52,192	65,314	0.109	1	Benzene, 1-ethyl-2-methyl-	94	120.094
**8**	7.006	216,109	60,348	72,722	0.086	1	Benzene, 1,2,4-trimethyl-	95	120.094
**9**	7.104	413,451	99,796	110,633	0.257	1	Benzene, 1,2,3-trimethyl-	97	120.094
**10**	7.441	64,100	15,801	26,583	0.092	1	Benzene, 1,3,5-trimethyl-	94	120.094
**11**	9.101	272,932	25,660	34,264	0.372	1	Benzene, 1-ethyl-2,3-dimethyl-	97	134.11
**12**	11.922	5662	3468	6417	0.063	1	Acetylcodein	10	341.163
**13**	13.129	168,105	21,107	23,862	0.292	1	Tetradecane	95	198.235
**14**	15.549	7168	3324	7210	0.069	1	3-(2-Methoxymethoxyethylidene)-2,2-dimethylbicyclo[2.2.1]heptane	12	210.162
**15**	16.064	38,886	11,461	14,980	0.12	1	Nonadecane	90	268.313
**16**	16.688	6749	2565	6265	0.074	1	Quinoline-5,8-dione-6-ol, 7-[[(4-cyclohexylbutyl)amino]methyl]-	32	342.194
**17**	19.846	15,534	5066	10,234	0.126	1	2,2-Dimethylpropanoic acid, 2,6-dimethylnon-1-en-3-yn-5-yl ester	10	250.193
**18**	20.11	336,957	94,797	100,901	0.172	1	Benzenepropanoic acid, 3,5-bis(1,1-dimethylethyl)-4-hydroxy-, methyl ester	93	292.204

## Data Availability

The original contributions presented in the study are included in the article, further inquiries can be directed to the corresponding authors.

## References

[1] Sung H., Ferlay J., Siegel R.L., Laversanne M., Soerjomataram I., Jemal A., Bray F. (2021). Global Cancer Statistics 2020: GLOBOCAN Estimates of Incidence and Mortality Worldwide for 36 Cancers in 185 Countries. CA Cancer J. Clin..

[2] Deo S.V.S., Sharma J., Kumar S. (2022). GLOBOCAN 2020 Report on Global Cancer Burden: Challenges and Opportunities for Surgical Oncologists. Ann. Surg. Oncol..

[3] GBD 2019 Colorectal Cancer Collaborators (2022). Global, regional, and national burden of colorectal cancer and its risk factors, 1990-2019: A systematic analysis for the Global Burden of Disease Study 2019. Lancet Gastroenterol. Hepatol..

[4] Xi Y., Xu P. (2021). Global colorectal cancer burden in 2020 and projections to 2040. Transl. Oncol..

[5] Almatroudi A. (2020). The Incidence Rate of Colorectal Cancer in Saudi Arabia: An Observational Descriptive Epidemiological Analysis. Int. J. Gen. Med..

[6] Alyabsi M., Algarni M., Alshammari K. (2021). Trends in Colorectal Cancer Incidence Rates in Saudi Arabia (2001–2016) Using Saudi National Registry: Early- Versus Late-Onset Disease. Front. Oncol..

[7] Kumar A., Gautam V., Sandhu A., Rawat K., Sharma A., Saha L. (2023). Current and emerging therapeutic approaches for colorectal cancer: A comprehensive review. World J. Gastrointest. Surg..

[8] Xie Y.-H., Chen Y.-X., Fang J.-Y. (2020). Comprehensive review of targeted therapy for colorectal cancer. Signal Transduct. Target. Ther..

[9] Shi J., Sun Z., Gao Z., Huang D., Hong H., Gu J. (2023). Radioimmunotherapy in colorectal cancer treatment: Present and future. Front. Immunol..

[10] Huang X., Yang Z., Xie Q., Zhang Z., Zhang H., Ma J. (2019). Natural products for treating colorectal cancer: A mechanistic review. Biomed. Pharmacother..

[11] Aiello P., Sharghi M., Mansourkhani S.M., Ardekan A.P., Jouybari L., Daraei N., Peiro K., Mohamadian S., Rezaei M., Heidari M. (2019). Medicinal Plants in the Prevention and Treatment of Colon Cancer. Oxid. Med. Cell Longev..

[12] Gomathinayagam R., Ha J.H., Jayaraman M., Song Y.S., Isidoro C., Dhanasekaran D.N. (2020). Chemopreventive and Anticancer Effects of Thymoquinone: Cellular and Molecular Targets. J. Cancer Prev..

[13] Khan A., Alsahli M.A., Aljasir M.A., Maswadeh H., Mobark M.A., Azam F., Allemailem K.S., Alrumaihi F., Alhumaydhi F.A., Alwashmi A.S.S. (2022). Safety, Stability, and Therapeutic Efficacy of Long-Circulating TQ-Incorporated Liposomes: Implication in the Treatment of Lung Cancer. Pharmaceutics.

[14] Khan A., Alsahli M.A., Aljasir M.A., Maswadeh H., Mobark M.A., Azam F., Allemailem K.S., Alrumaihi F., Alhumaydhi F.A., Almatroudi A.A. (2022). Experimental and Theoretical Insights on Chemopreventive Effect of the Liposomal Thymoquinone Against Benzo[a]pyrene-Induced Lung Cancer in Swiss Albino Mice. J. Inflamm. Res..

[15] Mostofa A.G.M., Hossain M.K., Basak D., Bin Sayeed M.S. (2017). Thymoquinone as a Potential Adjuvant Therapy for Cancer Treatment: Evidence from Preclinical Studies. Front. Pharmacol..

[16] Sadeghi E., Imenshahidi M., Hosseinzadeh H. (2023). Molecular mechanisms and signaling pathways of black cumin (*Nigella sativa*) and its active constituent, thymoquinone: A review. Mol. Biol. Rep..

[17] Li F., Qasim S., Li D., Dou Q.P. (2022). Updated review on green tea polyphenol epigallocatechin-3-gallate as a cancer epigenetic regulator. Semin. Cancer Biol..

[18] Negri A., Naponelli V., Rizzi F., Bettuzzi S. (2018). Molecular Targets of Epigallocatechin—Gallate (EGCG): A Special Focus on Signal Transduction and Cancer. Nutrients.

[19] Giordano A., Tommonaro G. (2019). Curcumin and Cancer. Nutrients.

[20] Weng W., Goel A. (2022). Curcumin and colorectal cancer: An update and current perspective on this natural medicine. Semin. Cancer Biol..

[21] Alrumaihi F.A., Khan M.A., Allemailem K.S., Alsahli M.A., Almatroudi A., Younus H., A Alsuhaibani S., Algahtani M., Khan A. (2021). Methanolic Fenugreek Seed Extract Induces p53-Dependent Mitotic Catastrophe in Breast Cancer Cells, Leading to Apoptosis. J. Inflamm. Res..

[22] El Bairi K., Ouzir M., Agnieszka N., Khalki L. (2017). Anticancer potential of Trigonella foenum graecum: Cellular and molecular targets. Biomed. Pharmacother..

[23] Promdam N., Panichayupakaranant P. (2022). [6]-Gingerol: A narrative review of its beneficial effect on human health. Food Chem. Adv..

[24] Nafees S., Zafaryab Md Mehdi S.H., Zia B., Rizvi M.A., Khan M.d.A. (2021). Anti-Cancer Effect of Gingerol in Cancer Prevention and Treatment. Anticancer Agents Med. Chem..

[25] Sharma S., Shukla M.K., Sharma K.C., Tirath Kumar L., Anal J.M.H., Upadhyay S.K., Bhattacharyya S., Kumar D. (2023). Revisiting the therapeutic potential of gingerols against different pharmacological activities. Naunyn Schmiedebergs Arch. Pharmacol..

[26] Yusof K.M., Makpol S., Fen L.S., Jamal R., Wan Ngah W.Z. (2019). Suppression of colorectal cancer cell growth by combined treatment of 6-gingerol and γ-tocotrienol via alteration of multiple signalling pathways. J. Nat. Med..

[27] Honari M., Shafabakhsh R., Reiter R.J., Mirzaei H., Asemi Z. (2019). Resveratrol is a promising agent for colorectal cancer prevention and treatment: Focus on molecular mechanisms. Cancer Cell Int..

[28] Kataria R., Khatkar A. (2019). Resveratrol in Various Pockets: A Review. Curr. Top. Med. Chem..

[29] Ahmadi R., Ebrahimzadeh M.A. (2020). Resveratrol—A comprehensive review of recent advances in anticancer drug design and development. Eur. J. Med. Chem..

[30] Efferth T. (2017). From ancient herb to modern drug: *Artemisia annua* and artemisinin for cancer therapy. Semin. Cancer Biol..

[31] Septembre-Malaterre A., Lalarizo Rakoto M., Marodon C., Bedoui Y., Nakab J., Simon E., Hoarau L., Savriama S., Strasberg D., Guiraud P. (2020). *Artemisia annua*, a Traditional Plant Brought to Light. Int. J. Mol. Sci..

[32] Tu Y. (2011). The discovery of artemisinin (qinghaosu) and gifts from Chinese medicine. Nat. Med..

[33] Su X.-Z., Miller L.H. (2015). The discovery of artemisinin and the Nobel Prize in Physiology or Medicine. Sci. China Life Sci..

[34] Juteau F., Masotti V., Bessière J.M., Dherbomez M., Viano J. (2002). Antibacterial and antioxidant activities of *Artemisia annua* essential oil. Fitoterapia.

[35] Lang S.J., Schmiech M., Hafner S., Paetz C., Steinborn C., Huber R., El Gaafary M., Werner K., Schmidt C.Q., Syrovets T. (2019). Antitumor activity of an *Artemisia annua* herbal preparation and identification of active ingredients. Phytomedicine.

[36] Appalasamy S., Lo K.Y., Ch’ng S.J., Nornadia K., Othman A.S., Chan L.-K. (2014). Antimicrobial Activity of Artemisinin and Precursor Derived from In Vitro Plantlets of *Artemisia annua* L. Biomed. Res. Int..

[37] Poiată A., Tuchiluş C., Ivănescu B., Ionescu A., Lazăr M.I. (2009). Antibacterial activity of some Artemisia species extract. Rev Med Chir. Soc. Med. Nat..

[38] Sen R., Bandyopadhyay S., Dutta A., Mandal G., Ganguly S., Saha P., Chatterjee M. (2007). Artemisinin triggers induction of cell-cycle arrest and apoptosis in Leishmania donovani promastigotes. J. Med. Microbiol..

[39] Rassias D.J., Weathers P.J. (2019). Dried leaf *Artemisia annua* efficacy against non-small cell lung cancer. Phytomedicine.

[40] Singh N.P., Lai H.C. (2004). Artemisinin induces apoptosis in human cancer cells. Anticancer Res..

[41] König M., von Hagens C., Hoth S., Baumann I., Walter-Sack I., Edler L., Sertel S. (2016). Investigation of ototoxicity of artesunate as add-on therapy in patients with metastatic or locally advanced breast cancer: New audiological results from a prospective, open, uncontrolled, monocentric phase I study. Cancer Chemother. Pharmacol..

[42] Wang C.-Z., Wan C., Luo Y., Zhang C.-F., Zhang Q.-H., Chen L., Liu Z., Wang D.H., Lager M., Li C.-H. (2022). Effects of dihydroartemisinin, a metabolite of artemisinin, on colon cancer chemoprevention and adaptive immune regulation. Mol. Biol. Rep..

[43] Zhang H., Zhou F., Wang Y., Xie H., Luo S., Meng L., Su B., Ye Y., Wu K., Xu Y. (2020). Eliminating Radiation Resistance of Non-Small Cell Lung Cancer by Dihydroartemisinin Through Abrogating Immunity Escaping and Promoting Radiation Sensitivity by Inhibiting PD-L1 Expression. Front. Oncol..

[44] Salaroli R., Andreani G., Bernardini C., Zannoni A., La Mantia D., Protti M., Mercolini L., Isani G. (2022). Anticancer activity of an *Artemisia annua* L. hydroalcoholic extract on canine osteosarcoma cell lines. Res. Vet. Sci..

[45] Ahuja A., Yi Y.-S., Kim M.-Y., Cho J.Y. (2018). Ethnopharmacological properties of Artemisia asiatica: A comprehensive review. J. Ethnopharmacol..

[46] Kamarya Y., Xia L., Li J. (2022). Chemical Constituents and Antitumor Mechanisms of *Artemisia*. Anticancer Agents Med. Chem..

[47] Allemailem K.S. (2022). Aqueous Extract of *Artemisia annua* Shows In Vitro Antimicrobial Activity and an In Vivo Chemopreventive Effect in a Small-Cell Lung Cancer Model. Plants.

[48] Posadino A.M., Giordo R., Pintus G., Mohammed S.A., Orhan I.E., Fokou P.V.T., Sharopov F., Adetunji C.O., Gulsunoglu-Konuskan Z., Ydyrys A. (2023). Medicinal and mechanistic overview of artemisinin in the treatment of human diseases. Biomed. Pharmacother..

[49] Verma S., Das P., Kumar V.L. (2017). Chemoprevention by artesunate in a preclinical model of colorectal cancer involves down regulation of β-catenin, suppression of angiogenesis, cellular proliferation and induction of apoptosis. Chem. Biol. Interact..

[50] Hamoya T., Fujii G., Iizumi Y., Narita T., Komiya M., Matsuzawa Y., Miki K., Kondo T., Kishimoto S., Watanabe K. (2021). Artesunate inhibits intestinal tumorigenesis through inhibiting wnt signaling. Carcinogenesis.

[51] Tong Y., Liu Y., Zheng H., Zheng L., Liu W., Wu J., Ou R., Zhang G., Li F., Hu M. (2016). Artemisinin and its derivatives can significantly inhibit lung tumorigenesis and tumor metastasis through Wnt/β-catenin signaling. Oncotarget.

[52] Tran K.Q., Tin A.S., Firestone G.L. (2014). Artemisinin triggers a G1 cell cycle arrest of human Ishikawa endometrial cancer cells and inhibits cyclin-dependent kinase-4 promoter activity and expression by disrupting nuclear factor-κB transcriptional signaling. Anticancer Drugs.

[53] El-Said K.S., Haidyrah A.S., Mobasher M.A., Khayyat A.I.A., Shakoori A., Al-Sowayan N.S., Barnawi I.O., Mariah R.A. (2023). *Artemisia annua* Extract Attenuate Doxorubicin-Induced Hepatic Injury via PI-3K/Akt/Nrf-2-Mediated Signaling Pathway in Rats. Int. J. Mol. Sci..

[54] Xia J., Dai Q., He S., Jia H., Liu X.-G., Hua H., Zhou M., Wang X. (2023). Artesunate alleviates 5-fluorouracil-induced intestinal damage by suppressing cellular senescence and enhances its antitumor activity. Discov. Oncol..

[55] Tcheng M., Ahmed N., Spagnuolo P.A. (2023). Structure defines bioactivity of avocado-derived acetogenins. Stud. Nat. Prod. Chem..

[56] Hati S., Tripathy S., Dutta P.K., Agarwal R., Srinivasan R., Singh A., Singh S., Sen S. (2016). Spiro[pyrrolidine-3, 3′-oxindole] as potent anti-breast cancer compounds: Their design, synthesis, biological evaluation and cellular target identification. Sci. Rep..

[57] Zhou Y., Zhou Z., Chan D., Chung P.Y., Wang Y., Chan A.S.C., Law S., Lam K.H., Tang J.C.O. (2022). The Anticancer Effect of a Novel Quinoline Derivative 91b1 through Downregulation of Lumican. Int. J. Mol. Sci..

[58] Li J., Duan M., Yao X., Tian D., Tang J. (2019). Prenylated benzenepropanoic acid analogues from the *Citrus grandis* (L.) Osbeck and their anti-neuroinflammatory activity. Fitoterapia.

[59] Nalin Pagi D.D., Payal Patel H.J. (2017). Antimicrobial Activity and Phytochemical Screening of *Aloe vera* (*Aloe barbadensis* Miller). Int. J. Curr. Microbiol. Appl. Sci..

[60] Alrumaihi F., Khan M.A., Babiker A.Y., Alsaweed M., Azam F., Allemailem K.S., Almatroudi A.A., Ahamad S.R., Alsugoor M.H., Alharbi K.N. (2022). Lipid-Based Nanoparticle Formulation of Diallyl Trisulfide Chemosensitizes the Growth Inhibitory Activity of Doxorubicin in Colorectal Cancer Model: A Novel In Vitro, In Vivo and In Silico Analysis. Molecules.

[61] Li J., Liu Z., Yu H., Xue Q., Qu X. (2020). Effects of artemisinin on proliferation and apoptosis of human liver cancer HepG2 cells. Medicine.

[62] Han X., Chai Y., Lv C., Chen Q., Liu J., Wang Y., Chou G. (2022). Sesquiterpenes from *Artemisia annua* and Their Cytotoxic Activities. Molecules.

[63] Guo S., Ma J., Xing Y., Shi L., Zhang L., Xu Y., Jin X., Yan S., Shi B. (2022). *Artemisia annua* L. Aqueous Extract Promotes Intestine Immunity and Antioxidant Function in Broilers. Front. Vet. Sci..

[64] Abate G., Zhang L., Pucci M., Morbini G., Mac Sweeney E., Maccarinelli G., Ribaudo G., Gianoncelli A., Uberti D., Memo M. (2021). Phytochemical Analysis Anti-Inflammatory Activity of Different Ethanolic Phyto-Extracts of *Artemisia annua*, L. Biomolecules.

[65] Zheng H., Lu Z., Wang R., Chen N., Zheng P. (2016). Establishing the colitis-associated cancer progression mouse models. Int. J. Immunopathol. Pharmacol..

[66] Kim H.S., Kundu J.K., Lee J.-S., Oh T.-Y., Na H.-K., Surh Y.-J. (2008). Chemopreventive Effects of the Standardized Extract (DA-9601) of *Artemisia asiatica* on Azoxymethane-Initiated and Dextran Sulfate Sodium-Promoted Mouse Colon Carcinogenesis. Nutr. Cancer.

[67] Khan A., Alhumaydhi F.A., Alwashmi A.S., Allemailem K.S., Alsahli M.A., Alrumaihi F.A., Almatroudi A., A Mobark M., Mousa A., A Khan M. (2020). Diallyl Sulfide-Mediated Modulation of the Fatty Acid Synthase (FASN) Leads to Cancer Cell Death in BaP-Induced Lung Carcinogenesis in Swiss Mice. J. Inflamm. Res..

[68] Corti A., Franzini M., Paolicchi A., Pompella A. (2010). Gamma-glutamyltransferase of Cancer Cells at the Crossroads of Tumor Progression, Drug Resistance and Drug Targeting. Anticancer Res..

[69] Samuel T.V., Garg A. (2020). Evaluation of serum adenosine deaminase and gamma glutamyl transferase in cancer cervix -A low-cost diagnostic tool. Int. J. Clin. Biochem. Res..

[70] Lamas B., Natividad J.M., Sokol H. (2018). Aryl hydrocarbon receptor and intestinal immunity. Mucosal Immunol..

[71] Thomas C.M., Sweep C.G. (2001). Serum tumor markers: Past, state of the art, and future. Int. J. Biol. Markers.

